# Prostate Cancer Screening in Jamaica: Results of the Largest National Screening Clinic

**DOI:** 10.1155/2016/2606805

**Published:** 2016-02-29

**Authors:** Belinda F. Morrison, William Aiken, Richard Mayhew, Yulit Gordon, Marvin Reid

**Affiliations:** ^1^Department of Surgery, University of the West Indies, Mona, Kingston, Jamaica; ^2^Jamaica Cancer Society, Kingston, Jamaica; ^3^Tropical Metabolism and Research Institute, University of the West Indies, Kingston, Jamaica

## Abstract

Prostate cancer is highly prevalent in Jamaica and is the leading cause of cancer-related deaths. Our aim was to evaluate the patterns of screening in the largest organized screening clinic in Jamaica at the Jamaica Cancer Society. A retrospective analysis of all men presenting for screening at the Jamaica Cancer Society from 1995 to 2005 was done. All patients had digital rectal examinations (DRE) and prostate specific antigen (PSA) tests done. Results of prostate biopsies were noted. 1117 men of mean age 59.9 ± 8.2 years presented for screening. The median documented PSA was 1.6 ng/mL (maximum of 5170 ng/mL). Most patients presented for only 1 screen. There was a gradual reduction in the mean age of presentation for screening over the period. Prostate biopsies were requested on 11% of screening visits; however, only 59% of these were done. 5.6% of all persons screened were found to have cancer. Of the cancers diagnosed, Gleason 6 adenocarcinoma was the commonest grade and median PSA was 8.9 ng/mL (range 1.5–1059 ng/mL). Older men tend to screen for prostate cancer in Jamaica. However, compliance with regular maintenance visits and requests for confirmatory biopsies are poor. Screening needs intervention in the Jamaican population.

## 1. Introduction

Prostate cancer is a major burden in Jamaica. It ranks as the commonest cancer in the island and is the leading cause of cancer-related deaths [1–3]. Between 1998 and 2002, the Jamaica Cancer Registry reported that 873 new cases of prostate cancer were diagnosed in the Kingston and St. Andrew region of Jamaica [1]. Kingston and St. Andrew represent approximately 26% of the population of Jamaica, which comprises 2.6 million persons. In 1999, there were 445 reported deaths from prostate cancer in Jamaica [2]. This figure represented 30% of all male cancer-related deaths and is the only available data published on cancer mortality in Jamaica. Advanced age, black race, family history, and dietary habits are significant risk factors for development of the disease. Widespread screening for prostate cancer has the potential to reduce the burden of metastatic disease and significantly reduce the mortality of the disease [4, 5]. Recently the relevance of prostate cancer screening has been questioned; however, the importance and relevance is undeniable in high-risk individuals (blacks and family history) [6].

The Jamaica Cancer Society has the largest and most organized screening clinic for prostate cancer to date in Jamaica. The Jamaica Cancer Society (JCS) is a nonprofit, nongovernmental organization, which was formed in 1955, engaged in activities for the prevention and control of cancer in Jamaica. The head office is located in Kingston with three branches in the parishes of St. Ann/St. Mary, Manchester, and St. Elizabeth. Screening services are provided by Volunteer Members of the Jamaica Urological Society. Recommendations for screening are annual visits commencing at age of 40 years. Males presenting for screening have digital rectal examinations (DRE) and prostate specific antigen (PSA). Men with abnormal PSAs and DREs are recommended to have confirmatory transrectal ultrasound (TRUS) guided biopsies. All men diagnosed with prostate cancer are referred for treatment at a urology clinic of their choice.

Jamaica lacks a formalized national governmental policy for prostate cancer screening. Hence, prior to the establishment of the JCS, screening was infrequent and largely opportunistic. The exact baseline rate of prostate cancer screening prior to the establishment of the JCS is unknown and to the best of our knowledge, this is the first study documenting prostate cancer screening in Jamaica.

We therefore sought to evaluate the patterns of screening in the largest organized screening clinic in Jamaica at the Jamaica Cancer Society from 1995 to 2005.

## 2. Materials and Methods

The study was a retrospective analysis of all male patients presenting for prostate cancer screening at the headquarters of the Jamaica Cancer Society, Kingston, Jamaica, from 1995 to 2005. The Jamaica Cancer Society, a nonprofit, nongovernmental organization began offering prostate cancer screening in Kingston, Jamaica, in 1995. The screening clinic is located in urban Jamaica, serving about 26% of the population. Screening is done voluntarily by local urologists (Members of the Jamaica Urological Society).

### 2.1. Study Population

The study population included men at least 40 years of age. All men had a DRE and PSA test requested at each screening visit. Men who had abnormal DRE or PSA results were recommended to have a confirmatory TRUS guided biopsy of the prostate. Men who had nonmalignant histopathology reports after TRUS guided biopsies of the prostate were advised to be reviewed early (6 weeks to 6 months) and on occasion repeat biopsies were recommended. Men who had normal DRE and PSA results were recommended to return in 1 year for repeat screening. All males who were diagnosed with prostate cancer were referred to an external urology outpatient facility for further treatment.

### 2.2. Variables

Medical records of all eligible screened men were reviewed. Patient age, DRE findings, and PSA results at each screening visit were recorded. All requested prostate biopsies and histopathology results, including Gleason grade of reported cancers, were recorded.

### 2.3. Statistical Analysis

Summary values were expressed as counts, mean ± s.d., or medians as appropriate. Several PSA values were reported as <1.5 ng/mL. PSA values were therefore censored at 1.5 ng/mL and these values were included. Summary measure for PSA was expressed as medians and inference done by nonparametric means. Data were analyzed using Stata 12 for Windows (College Station, USA).

## 3. Results

### 3.1. Patient Characteristics

1117 men of mean age 59.9 ± 8.2 years were screened during the study period. The total number of screening visits was 2281. Median PSA was 1.6 ng/mL with a maximum of 5170 ng/mL.

### 3.2. Screening Visits

**Figure 1:**
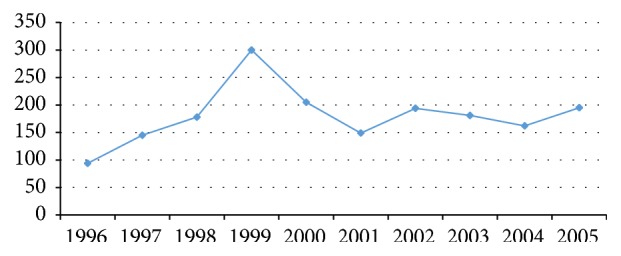
Number of screening visits at the Jamaica Cancer Society per year (1995–2005).

**Figure 2:**
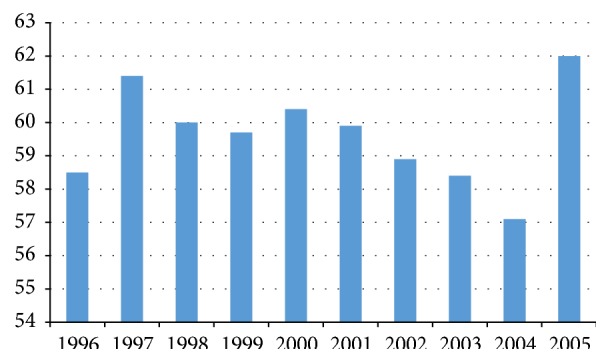
Variation in mean age (years) of patients presenting for biopsy.

The mean number of patient visits per calendar year was 180, with a peak of 300 men screening in 1999 (Figure 1). There was no significant change in the mean screening age between 1995 and 2005 (Figure 2). 30%, 17%, and 13% of screened patients presented for only 1, 2, and 3 visits, respectively, during the study period.

### 3.3. TRUS Prostate Biopsy

TRUS prostate biopsies were recommended on 240 occasions (11% of screening visits). Only 59% of persons complied with recommended TRUS prostate biopsies. Prostate adenocarcinoma was diagnosed in 5.6% of screened men (63 men). 54% of men diagnosed with adenocarcinoma had Gleason 6 grade. The median PSA of men diagnosed with cancer was 8.9 ng/mL (range 1.5–1059 ng/mL).

Table 1 displays the histopathology findings in men whose reports were nonmalignant. Significant inflammation and atypical small acinar proliferation (ASAP) were reported in 12% and 6% of nonmalignant reports, respectively.

## 4. Discussion

The mission statement of the Jamaica Cancer Society is to eliminate cancer as a major health problem in Jamaica. The society has therefore established prostate cancer screening clinics to achieve this mission. Screening services have also been implemented in rural parishes in Jamaica, St. Ann, Manchester, and St. Elizabeth, to extend these efforts. The efforts of volunteers and the society in increasing public awareness about prostate cancer aim to increase screening by high-risk men. We found that most men who presented for screening did not comply with medical recommendations for maintenance of annual screening visits or confirmatory TRUS biopsies. We also found that the mean age of men presenting for prostate screening was much older than recommended by the Jamaica Urological Society and the American Urological Association for high-risk males.

Men of African ethnicity are well known to be of high risk for developing prostate cancer. In the USA this ethnic disparity is demonstrated with African-American men having a 60% higher incidence rate and 2-3 times greater mortality rate of prostate cancer than Caucasian-Americans [7]. In the United Kingdom, both African and Caribbean descent black men have similar higher rates of prostate cancer compared to white men [8]. Incidence rates of prostate cancer in several Caribbean islands with a predominantly black population are also notably high. Prostate cancer is the leading cancer in Barbados, with an incidence rate of 160.4 per 100,000 but with a high mortality rate of 63.2 to 101.6 per 100,000 [9]. The prevalence of prostate cancer in Tobago in men between the ages of 40 and 79 years was found to be 10%, 3-4-fold higher than studies from predominantly Caucasian populations [10]. Data on the epidemiology of prostate cancer in Western African countries is sparse due to absence of cancer-registries and resource constraints. It appears that the shared common increased prostate cancer risk among men of African descent appears to be genetically related [11]. Environmental factors such as diet, inflammation, and obesity may impact these genes and alter risk [12–14].

We believe that our results reveal common barriers to screening in the Jamaican population. Several studies have reported that African-American and Afro-Caribbean men are less likely to screen for prostate cancer than other racial groups [15, 16]. The poor compliance with regular screening and recommendations for prostate biopsies were unexpected in men who presented voluntarily to the Jamaica Cancer Society for screening. However, fear and anxiety related to a possible unfavourable diagnosis of prostate cancer could account for poor compliance. Fear of treatment related adverse effects if prostate cancer is diagnosed may also affect maintenance patterns of screening. Anxiety related to the perceived embarrassing nature of the DRE has also been linked to screening barriers in African-American men [17]. Lee et al. reported that African-American, Jamaican, and Trinidadian black men were less likely to undergo DRE screening and 74–84% were less likely to maintain DRE screening compared to whites [18]. The Jamaica Health and Lifestyle Survey 11 reported that 79.2% of sampled Jamaican men never had a DRE [19]. Lee et al. reported that Afro-Caribbean men who perceived the healthcare system as inconvenient or found reliable care inaccessible were less likely to participate in screening [20]. In fact Holmes reported that a longer distance of patient's home to the urologist's office affects access to care and disproportionately affects black men screening habits [21]. Other reported barriers to screening have been low socioeconomic status, lack of insurance, and low prostate cancer knowledge content [18]. The Jamaica Health and Lifestyle Survey reported that men with a higher socioeconomic status and higher level of education were more likely to have undergone a DRE. Poor physician-patient interaction may also serve as a barrier to prostate cancer screening. Men who do not have consistent health care provider are less likely to initiate or maintain screening, since health care providers would provide health education and counselling about screening [22]. We believe that the high mean age of screening is a reflection of the multiple barriers to prostate cancer screening.

We consider the prevalence rate of 5.6% of prostate cancer diagnosed at the Jamaica Cancer Society considerably lower than expected. 11% of screened men had abnormal screening tests; however, only 59% of these had confirmatory prostate biopsies. In addition, many men did not present for repeat screening visits so several cases of cancer may have been missed. We believe that 5.6% may be an underestimate of the true prevalence rate of prostate cancer discovered in the screened sample.

We acknowledge that a limitation of the study is its retrospective nature and our inability to determine the outcome of those men who defaulted from follow-up screening. We are also aware that the sample of men screened at the Jamaica Cancer Society may represent a selection bias due to the clinic's location and socioeconomic status of those attending.

Screening patterns for prostate cancer in Jamaican men reveal that barriers exist which prevent compliance. Strategic action plans by the local health ministry to promote a national screening programme and a national health education programme must be implemented for effective screening. Continued work of nongovernmental agencies such as the Jamaica Cancer Society and service clubs, as well as advocacy by volunteers and survivors, may be effective in changing screening patterns. In addition, engagement of organizations such as the church, community, and support of the family unit has been proven to be effective.

## 5. Conclusions

Prostate cancer was detected in 5.6% of men screened at the Jamaica Cancer Society. Older men tended to screen for prostate cancer and compliance with regular maintenance visits and requests for confirmatory biopsies were poor.

## Figures and Tables

**Table 1:** Nonmalignant histopathology results of prostate biopsies at the Jamaica Cancer Society (1995–2005).

Histopathology findings	*N* (%)
Negative	62 (78)
HGPIN	3 (4)
ASAP	5 (6)
Inflammation	9 (12)

HGPIN: high grade prostatic intraepithelial neoplasia.

ASAP: atypical small acinar proliferation.
